# Pulmonary Metastases from an Undifferentiated Embryonal Sarcoma of the Liver: A Case Report and Review

**DOI:** 10.1155/2018/7840865

**Published:** 2018-09-04

**Authors:** Mingxia Shi, Hongzhi Xu, Guillermo P. Sangster, Xin Gu

**Affiliations:** ^1^Department of Pathology and Translational Pathobiology, Louisiana State University Health Science Center-Shreveport, 1501 Kings Highway, Shreveport, LA 71130, USA; ^2^Department of Radiology, Louisiana State University Health Science Center-Shreveport, 1501 Kings Highway, Shreveport, LA 71130, USA

## Abstract

Undifferentiated embryonal sarcoma of the liver (UESL) is a rare malignant hepatic tumor that occurs primarily in children. Only a limited number of cases have been reported in the literature due to low incidence of one per million, and reports of metastatic lesion of UESL are even rarer. We hereby describe the case of a 13-year-old male who presented with a palpable mass with imaging findings suggestive of a large complex tumor in the right lobe of the liver. He underwent extended right hepatectomy followed by adjuvant chemotherapy. The tumor was confirmed to be UESL by postoperative pathology and immunohistochemical staining analysis. Four years later, surveillance imaging revealed a small lung nodule in the left lower lobe. Complete removal of the lung tumor by wedge resection was performed, and a histological diagnosis of metastatic UESL was made. The patient also received postoperative adjuvant chemotherapy and is currently in a good general condition and tumor-free in the present eight-month period. This case is presented with emphasis on clinicopathological and immunohistochemical findings of the primary UESL and lung metastases with the aim of collecting more data and expanding our understanding of this rare malignancy.

## 1. Introduction

Malignant liver tumors represent approximately for 1% to 4% of all solid tumors in children [1]. Undifferentiated embryonal sarcoma of the liver (UESL), first described by Stocker and Ishak in 1978 as a rare aggressive mesenchymal tumor of the liver [2], accounts for approximately 9–13% of all childhood malignant hepatic tumors [3] and is the third most common hepatic malignancy in children after hepatoblastoma and hepatocellular carcinoma. It occurs predominantly in children, with a peak incidence between 6 and 10 years of age [2]. It has also infrequently been reported in adults. Due to its low incidence of one per million [4], only a limited number of cases have been reported in the literature.

Diagnosis of UESL relies on postoperative pathological examination and immunohistochemical results. It is based on the tumor morphology, patient's age, and tumor location (primary hepatic mass) together with a panel of undifferentiated pathologic markers including vimentin, desmin, CD10, CD68, alpha1-antitrypsin, and ruling out of other pathologies. In the past, prognosis of UESL had been poor due to local recurrence, tumor rupture, and metastatic disease [2, 5]. More recently, aggressive treatment regimens that combine complete surgical resection of the hepatic tumor and effective multiagent chemotherapy have improved survival substantially [6, 7]. Although metastases of UESL have been reported to occur in 5–13% of children [8, 9], only very rare cases of metastatic UESL, which mostly are present at time of diagnosis, have been reported in the literature [4, 10, 11]. The optimal treatment for metastatic UESL has not been defined, likely due to the rarity of the disease and a paucity of data.

Herein, we report a case of UESL found in a 13-year-old male who developed a lung metastasis four years after hepatectomy and adjuvant chemotherapy and highlight the clinicopathological and immunohistochemical features of the primary and metastatic lesion.

## 2. Case Presentation

**Figure 1:**
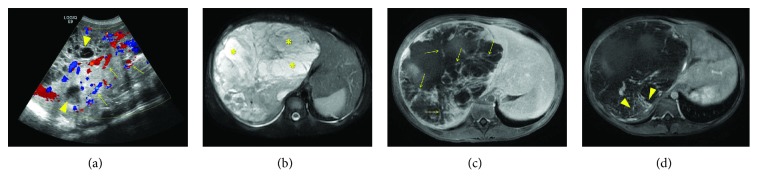
(a) Ultrasonography of the liver. Grayscale with Doppler image demonstrates a partially defined hepatic mass with multiple internal cystic foci (arrowheads) and increased intralesional vascularization (arrows). MRI of the abdomen with axial T2 fat saturation (b) and LAVA (liver acquisition with volume acceleration sequence) after intravenous contrast administration at 5 min (c) and 20 min (d) show a large heterogeneous mass within the right hepatic lobe. A predominant intralesional cystic/necrotic component (^∗^), multiple internal septations (arrows), and residual solid tissue (arrowheads) are seen.

**Figure 2:**
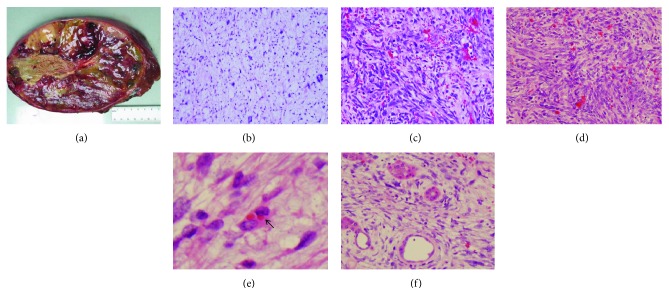
Pathological findings of the UESL. (a) Macroscopic appearance of the tumor. Histological examination showed (b, c, and d) hypocellular myxoid and hypercellular areas containing stellate and spindle-shaped malignant cells with scattered bizarre multinucleated giant cells, (e) the presence of cytoplasmic eosinophilic hyaline globules (arrow), and (f) cords of hepatocytes and bile ducts entrapped within the tumor (hematoxylin and eosin stain; original magnification: (b) ×100; (c, d, and f) ×200; (e) ×400).

**Figure 3:**
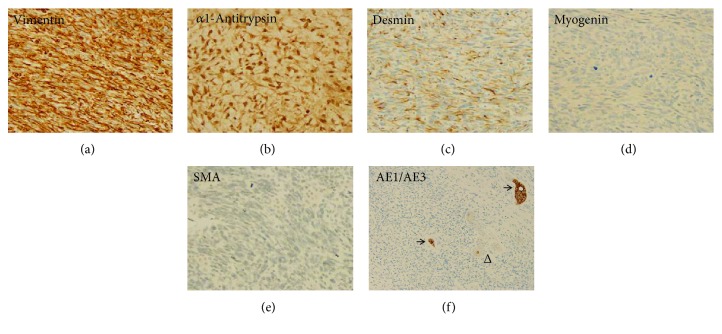
Immunohistochemical staining of the tumor cells in the UESL showed strong positivity for vimentin (a) and *α*1-antitrypsin (b), patchy positivity for desmin (c), and negative for myogenin (d), smooth muscle actin (SMA) (e), and pancytokeratin AE1/AE3 (f). The entrapped hepatic cords were negative for AE1/AE3 (f) (arrowhead), but AE1/AE3 highlighted the entrapped bile ducts (f) (arrow) (immunoperoxidase; original magnification: ×200).

A 13-year-old male was transferred to our Pediatric Hematology/Oncology Clinic for evaluation of a large liver mass detected by an abdominal computed tomography (CT) scan in an outside hospital. He presented with increasing abdominal distension of several months' duration and denied fever, abdominal pain, nausea, vomiting, or loss of appetite. During his admission, a physical examination revealed that the liver edge was palpable 6 cm below the right costal margin and no abdominal tenderness or guarding was present. Laboratory investigations demonstrated slightly elevated lactate dehydrogenase (263 U/L, normal range: 74–250 U/L). His blood count, liver function tests, and other liver enzymes as well as serum alpha-fetoprotein (AFP) were within normal range. Ultrasonography revealed a partially defined hepatic mass with multiple internal cystic foci, and an increased intralesional vascularization is identified (Figure 1(a)). Magnetic resonance imaging (MRI) of the abdomen revealed a 17 × 18 × 20 cm heterogeneous predominantly cystic mass with thick internal septations, residual solid tissue, and peripheral neovascular formation in the right hepatic lobe (Figures 1(b)–1(d)). Extended right hepatectomy was performed. Intraoperative frozen section was submitted with interpretation of malignant neoplasm. Grossly, the resected specimen consisted of a 19.5 × 14 × 16 cm well-circumscribed mass with a fibrous pseudocapsule. Cut surface of the tumor showed a variegated appearance of gray, solid glistening tumor alternating with soft gelatinous areas with dark-brown and yellow-green areas of hemorrhage and necrosis (Figure 2(a)). On microscopic examination, the tumor contains alternating hypocellular myxoid areas and hypercellular areas. It was comprised predominantly of pleomorphic cells that are spindle, oval, or stellate shaped and distributed in a fibrous or myxoid stroma (Figures 2(b)–2(d)). Some areas showed fibroblast-like fascicles and bundles. Focally, tumor cells were highly bizarre, with occasional large anaplastic multinucleated giant cells. Atypical mitotic figures were easily identified. Few sharply defined eosinophilic hyaline globules in the tumor cell cytoplasm were observed (Figure 2(e)). Entrapped bile ducts and hepatic cords were present in areas at the periphery of the tumor (Figure 2(f)). By immunohistochemistry, tumor cells stained positively for vimentin and alpha1-antitrypsin, partially positive for desmin, and negative for myogenin, smooth muscle actin (SMA), and pancytokeratin (AE1/AE3) (Figures 3(a)–3(f)). The AE1/AE3 stain highlighted the entrapped bile ducts (Figure 3(f)). The surgical margin was free. On the basis of these findings, a pathological diagnosis of UESL was made. Postoperative positron emission tomography (PET) scan did not reveal residual or metastatic disease. A five-month course of chemotherapy (VAdrC/VIE) including vincristine, doxorubicin, cyclophosphamide, ifosfamide, and etoposide was received, starting at 4 weeks after the operation, and he tolerated the chemotherapy well. The patient has been followed with imaging studies, including a whole-body PET scan.

**Figure 4:**
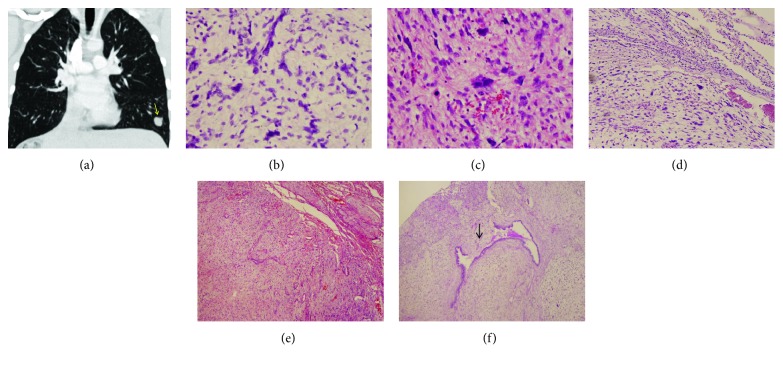
Pulmonary metastatic lesion of UESL. (a) CT of the thorax showed a well-defined solid nodular lesion (arrow) in the left lower lobe. (b, c) Histological examination of the lung lesion. (d) The uninvolved pulmonary parenchyma (to upper right of the center) is compressed. (e, f) Bronchioles entrapped within and at the periphery of the tumor. An isolated bronchiole is surrounded and focally destroyed by the infiltrating tumor (f) (arrow) (hematoxylin and eosin stain; original magnification: (f) ×40; (d) and (e) ×100; (b) and (c) ×200).

**Figure 5:**
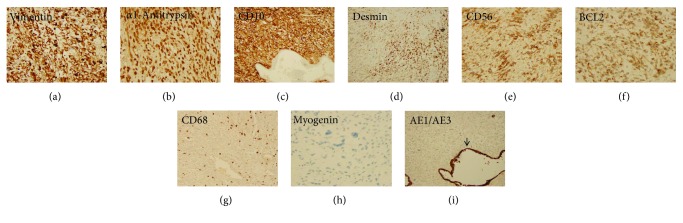
Immunohistochemical staining of the pulmonary metastatic lesion showed strong and diffuse positivity for vimentin, *α*1-antitrypsin, and CD10 (a–c), patchy positivity for desmin, CD56, and BCL2 (d–f), rare staining for CD68 (g), and negative for myogenin and AE1/AE3 (h and i). The entrapped bronchiolar epithelium was highlighted by AE1/AE3 (i) (arrow) (immunoperoxidase; original magnification: (c, d, g, and i) ×100; (a, b, e, f, and h) ×200).

At 48 months of follow-up, surveillance MRI showed a hyperintense, 7 mm lung lesion on T2-weighted images but PET scan was negative. Chest CT imaging at 50 months following the hepatectomy revealed a 1.7 × 1.4 cm lung nodule in the left lower lobe (Figure 4(a)) with no pleural or pericardial effusions. There was left hepatic lobe hypertrophy with no evidence of local tumor recurrence. The patient underwent lateral thoracotomy with wedge resection of the left lower lobe nodule. Macroscopically, the resected specimen consisted of a well-demarcated mass measuring 1.5 × 1.2 × 0.9 cm with soft and gelatinous cut surface. Histopathological studies revealed that the tumor was composed of pleomorphic stellate and spindled neoplastic cells in a predominantly myxoid matrix; scattered bizarre multinucleated giant cells and atypical mitotic figures were frequently seen (Figures 4(b)–4(d)). No evident intracellular or extracellular eosinophilic hyaline globules were observed. There were bronchioles entrapped within and at the periphery of the tumor (Figures 4(e) and 4(f)). Few isolated bronchioles were focally destroyed by the infiltrating tumor. Background lung parenchyma revealed atelectasis and marked vascular congestion. By immunohistochemistry, the tumor cells were strongly and diffusely positive for vimentin, *α*1-antitrypsin, and CD10, patchy positive for desmin, CD56, and BCL2, rare staining for CD68, and negative for myogenin and AE1/AE3 (Figures 5(a)–5(i)). The entrapped bronchiolar epithelium was highlighted by AE1/AE3 (Figure 5(i)). The pathological findings are consistent with metastases of the UESL. A chemotherapy regimen with olaratumab plus doxorubicin was received. At the time of this report, he is 9 months after wedge resection and remains well with no evidence of tumor recurrence.

## 3. Discussion

Undifferentiated embryonal sarcoma of the liver (UESL) is a relatively new distinct clinicopathologic entity that describes a rare malignancy arising from the primitive mesenchymal tissue of the liver [2]. It is mainly seen in young children and adolescents without gender predilection. UESL is rare among adults, with a female preponderance [12]. It represents fewer than 1% of all primary liver neoplasms in adults [13].

Patients with UESL usually have variable and nonspecific symptoms, with abdominal pain and abdominal mass reported to be the most common presenting complaints [14]. Other complaints, such as fever, nausea, vomiting, weight loss, fatigue, anorexia, and jaundice, may be presented. Spontaneous rupture resulting in intraperitoneal hemorrhage due to rapid tumor growth has also been reported [15]. There are no distinctive laboratory findings for UESL. Mild leukocytosis or leukopenia, low albumin, anemia, and slightly elevated transaminase levels and erythrocyte sedimentation rates may be seen. Evaluation of some tumor markers including AFP, cancer antigen 19-9, and carcinoembryonic antigen often yields normal results, but rare cases with increased levels of AFP and cancer antigen 125 have been reported [16]. There is one reported case of UESL that secretes erythropoietin, which was used as a marker of the tumor recurrence [17]. Our patient presented with asymptomatic abdominal mass and unremarkable laboratory findings, which is similar to what have been reported previously in most cases of childhood UESL.

The results of imaging studies of UESL are often nonspecific and inconclusive. On ultrasound (US) imaging, UESL usually appears as a hypoechoic solid mass. CT and MRI scans typically demonstrate a large mass with cystic attenuation. UESL is occasionally misdiagnosed as a benign hepatic lesion based on the cystic appearance seen on CT and MRI. This diagnostic pitfall may cause delayed management. There have been several case reports of UESL being mistaken for hydatid disease [18, 19]. Discrepancy of internal architecture on US and CT was considered one of the important characteristics of UESL. Such discordant or inconsistent imaging findings of a large hepatic lesion that has a seemingly cystic appearance on CT or MRI and a predominantly solid appearance on ultrasound should raise suspicion for this tumor [20, 21]. On angiography, UESL is most often hypovascular; however, avascular and hypervascular appearances have been reported [22]. Our case showed consistent images of the multicystic hepatic mass on US and CT. These characteristic imaging patterns account for the increased water content within the abundant myxoid stroma of UESL.

Preoperative diagnosis of UESL is challenging due to the lack of characteristic clinical manifestations and tumor markers, nonspecific radiological imaging, and the rarity of the disease. Definitive diagnosis relies on postoperative pathological examination and immunohistochemical results. UESL usually occurs as a large (10–30 cm), solitary well-circumscribed mass that is mostly localized in the right lobe of the liver, while it rarely develops in the hepatic left lobe or the bilateral lobes. The mass often has a fibrous pseudocapsule with compressed liver parenchyma. Cut surface reveals a heterogeneous appearance that is predominately solid but often has foci of cystic or gelatinous degeneration. Hemorrhagic and necrotic areas are common [19, 22–24]. Microscopically, UESL consists of medium to large sized spindle, oval, or stellate shaped pleomorphic cells that may be arranged either compactly or loosely in abundant myxoid matrix or fibrous stroma. Multinucleated giant cells, bizarre cells, and atypical mitosis are often seen. Trapped hepatocytes and bile duct cells can be observed at peripheral area of the tumor. Variable sized eosinophilic globules can be seen in the tumor cell cytoplasm and extracellular matrix [19, 22–26]. These hyaline globules are diastase-resistant and periodic acid-Schiff- (PAS-) positive and correspond with the prominent electron-dense complexes under an electron microscope [22]. The histopathologic characteristics of UESL in our case are similar to those described in the previous reports. Also in the literature, focal osteoid picture was reported in one adult case [26]. Extramedullary hematopoiesis has been noted in some of the cases [2, 27].

Immunohistochemically, the staining pattern of UESL is variable and nonspecific. The divergent staining or combined expression of fat, muscle, histiocytic, and epithelial markers suggests the origin of primitive mesenchymal stem cell, which may display partial differentiation. Usually, multiple immunostains are performed to help with the diagnosis as they also facilitate the exclusion of other tumors in the differential diagnosis, which includes poorly differentiated or sarcomatoid hepatocellular carcinoma, embryonal rhabdomyosarcoma, and other sarcomas. Tumor cells of UESL are consistently positive for vimentin and *α*1-antitrypsin. There is variable staining for desmin, smooth-muscle actin, CD68, CD56, BCL2, and CD10. No immunoreactivity has been described for HepPar-1, myogenin, CD34, CD117, S-100, Alk-1, or AFP [22–25]. Glypican 3 (GPC3) and paranuclear dot-like staining for cytokeratin has been reported [14, 27]. The immunohistochemical profiles in our case are consistent with a diagnosis of UESL.

In the past, prognosis of UESL had been poor; initial reports described mortality within 12 months of diagnosis, and the long-term disease-free survival rate was less than 37% [2, 5]. Poor prognosis of UESL is associated with local recurrence, tumor rupture, and its metastasis to other parts of the body. Since the widespread use of multimodal therapy, including primary resection, neoadjuvant or adjuvant chemotherapy, and radiation, the long-term survival rate of UESL patients has improved significantly and is currently reported to be >70% [4, 6, 7]. Currently, complete resection of hepatic tumor, combined with adjuvant chemotherapy, appears to be the mainstay of treatment. The chemotherapy regimens reported in the literature are varied as no standard regimens designed specifically for UESL. In addition, liver transplantation has been reported to improve survival for refractory, unresectable, or recurrent tumors [4, 6, 7].

Pulmonary metastases are a common manifestation of sarcoma. The pulmonary arteries are the most common route for metastases. Tumors most likely to metastasize to the lungs include those with a rich vascular supply draining directly into the systemic venous system. Metastases of UESL have been reported to occur in 5–13% of children [8, 9], and metastatic sites such as the lung, adrenal gland, peritoneum, and pleura have been reported [4, 10, 11, 28]. The tumor cells might spread hematogenously, via lymphatics or by direct extension. Tumor may also show direct involvement of the heart, with inferior vena cava tumor extension to the right atria [27]. The cases of metastatic UESL that reported in the literature are mostly present at the time of primary diagnosis. Plant et al. reported one patient with UESL recurred 2 years from diagnosis with bilateral paraspinal masses [28]. Our case reported an interval of 4 years between primary tumor treatment and development of lung metastasis. The mechanism of lung metastases occurring years after curative resection remains to be elucidated.

The optimal treatment of patients with metastases remains controversial. Some cases have shown that surgical resection combined with chemotherapy appears to be the most beneficial treatment strategy [8]. Xie et al. reported a case of UESL with lung metastasis in which they have achieved a good result using immunotherapy [10]. However, only rare cases of metastatic UESL have been reported in the literature, and knowledge of the metastatic lesions and its optimal treatment is tempered by the few cases available and large amounts of missing data.

## 4. Conclusions

Although current aggressive multimodal therapy is associated with favorable outcomes in children with UESL, intensive surveillance and follow-up for early detection of metastases is crucial to increase the chances of long-term survival. The patient reported herein developed a lung metastasis four years after hepatectomy and adjuvant chemotherapy. This case is presented with emphasis on clinicopathological and immunohistochemical findings of the primary UESL and lung metastases with the aim of collecting more data and expanding our understanding of this rare malignancy. Pulmonary metastasectomy for the isolated lung metastases of UESL with adjuvant chemotherapy may provide a reasonable long period of survival.
